# Progress in Phototherapy

**Published:** 1902-12-06

**Authors:** 


### Progress in Phototherapy.

(Concluded from page 146.)

Berry Hart [13] speaks well of X-rays as a good treatment for obliterating intractible sinuses. Williamson Pugh [14] speaks of the beneficial result of the rays being continued for long after the treatment is suspended. Speaking of radiography, Espina y Capo states that the shadow of the heart on an X-ray screen is smaller and lower down than the clinical dulness.

A useful localiser for X-ray work has been introduced by Wishart Little.[15] It takes the form of a sliding caliper scale, graduated for a fixed position of the tube and distance of that tube from the patient. Two photographs are taken on the same plate, the tube being moved from the first point 2 inches to one side of a vertical fixed point to the second position 2 inches to the other side of the same point. The distance of the tube from the patient is 15 to 20 inches, according to the thickness of the body. In using the calipers the fixed jaw rests upon one image and the movable one on the other, the distance of the object from the surface being read off the scale. If suitable allowances are made for other distances than 15–20 inches, or for moving more than 2 inches either way of the central vertical point, use can be made of the scale for any position required.

At the German Medical Congress [16] Professor Bie spoke on the action of light upon the skin. He observed that there were two factors, the heat and chemical properties of light. Finsen, he says, observed first a capillary dilatation, but finds no change in the blood, in the number of corpuscles or quantity of hæmoglobin. Bie thinks there is no evidence of increased metabolism, there being no increase of carbonic acid excretion. Finsen has shown that the embryos of lower animals prefer to rest under red light rather than other colours. Red and yellow are physically stimulating, while blue and green are depressing. Experiments show that human tissues are very permeable to light of all kinds. The blue and violet are active in bactericidal powers, which are due to the chemical properties of the rays. No light, however, can be powerful enough to reach the internal organs such as the lungs. Vesicles of small-pox have been modified by red light alone. In measles a French observer noticed that it checked the rash, though not the disease. On exposure to white light again the rash came out more fully. No definite results have been noticed in eczema with the chemical rays. Incandescent light is so poor in chemical rays that it is very doubtful if any good is to be expected from the light baths. The effect in lupus is half chemical, half bactericidal. Six hundred and fifty cases of lupus, treated by Finsen before 1900, showed 85 per cent. absolute benefit. Acne is improved, but not so much as lupus. Early cases of lupus, 10–20 years, are the most amenable to treatment.

Rumpel,[17] of Hamburg, advocates the incandescent light bath, even as a vapour bath, on account of its rapid action. Erysipelatous patients can submit longer than patients with healthy skins.

Hollander [18] finds no benefit in the light cure for lupus erythematosus. He thinks it a disease of the glands of the skin, and treats it successfully with enormous doses of quinine and the immediate application of iodine.

Suprarenal extract has been recommended by Allan Jamieson [19] as a useful adjunct to the light treatment of lupus. This blanches normal skin (1 in 1,000 on cotton wool for 10–15 mins.), though not so much as mucous membranes. The lupus deposits show up as through a diascope. He tried to remove skin over the deposits by Beiersdorf's salicylic acid and creosote plaster muslin, but the effect did not warrant its use.

Cases [20] are reported of acne rosacea, psoriasis, acne vulgaris, condylomata and syphilitic ulcerations, by means of the "Dermo" lamp combined with other treatment where necessary.

Mr. F. B. Aspinall [21] asks a few most important questions, and gives some valuable personal experience on the subject of electrical stimulation of the human body.

(1) Is everyone equally susceptible to an electric shock? He holds that there are great differences of resistance in different individuals, and also in the same individual at different times. The factors to be taken into account are many, viz.:—Moistures, callosity, and condition of skin; nervous temperament, the path which the current takes through the body, health or disease, drunkenness or sobriety, whether the person is awake or asleep, area, excellence or nature of contact, familarity with shock, etc.

(2) That persons differ in resistance according to their condition of health would seem indicated from the instance in which a person suffering from a renal complaint offered much less resistance than others who were healthy. Also, it is a recognised fact that persons of weak intellect can stand a much greater shock than the mentally sound. The susceptibility would seem to depend upon the nature of the disease.

(3) Does the physiological condition in which the patient is in when the shock is received make any difference? The moister the skin is the more likelihood of the current being shunted. A 2,000-volt shock passed through a man who was doing heavy work which made him sweat freely; it did not make him even insensible. A drunk man has been known to take a 2,000-volt shock through him without a fatal effect. Persons asleep have been strongly shocked without serious result.

(4) Does the path which the current takes through the body have any effect as regards the shock proving fatal? Different sensations are caused according to the path the shock takes in the body, and there is a distinct indication that certain paths are more dangerous than others. The left side, for instance, more dangerous than the right.

(5) Does the question of contact made, and whether burning takes place or not, have any effect upon the fatality of a shock? There is a great difference in individuals normally—the condition of moistness of the skin has much to do with the result. If the area is small, there is more risk of burning. Burning seems a provision of nature to render a point of contact less conducting.

(6) A man can receive a fatal shock without giving the "cry," and can even speak after having received a fatal shock.

(7) The after effect of a direct current is in his experience worse than that of an alternating one, considering the actual voltage, independent of the nature of supply, required to produce death. Below 600 volts the conditions must be abnormally favourable; below 1,000, favourable, while above 1,000 the higher the voltage the more easily the conditions are obtained necessary to cause death. He believes it is mainly a question of voltage whether anyone gets killed or not in either system of current, and of whether other circumstances are favourable. He asks :—(*a*) A more certain ready test as to whether a man is dead or not. (*b*) Whether something cannot be done when a man receives a shock to restore him. He thinks that holding a man's head downwards is beneficial as in restoring functions during anæsthesia.

Batelli[22] states that currents of high tension produce death by inhibiting the great nervous centres and causing cessation of respiration, while those of low tension lead to fibrillary contractions of the heart and so death. If this fibrillary tremor is present, no treatment will be of avail. Danger begins with alternating currents of 400-500 volts or continuous of 1,500 volts.

[13] Ibid., May 31. [14] Ibid., April 12. [15] Lancet, June 21. [16] Brit. Med. Jour., May 3, 1902. [17] Ibid. [18] Ibid. [19] Ibid., June 21. [20] Archiv. für Licht Therapie, April 1902. [21] Lancet, March 8. [22] Brit. Med. Jour. Oct. 11.

